# Serum microRNAs as peripheral markers of primary aldosteronism

**DOI:** 10.3389/fendo.2025.1511096

**Published:** 2025-03-20

**Authors:** Nikita Makhnov, Fredrik Axling, Elham Barazeghi, Peter Stålberg, Tobias Åkerström, Per Hellman

**Affiliations:** ^1^ Department of Surgical Sciences, Uppsala University, Uppsala, Sweden; ^2^ Department of Surgery, Karlstad Central Hospital, Karlstad, Sweden; ^3^ Center for Clinical Research and Education, Region of Värmland, Karlstad, Sweden; ^4^ Department of Surgery, Uppsala University Hospital, Uppsala, Sweden

**Keywords:** hyperaldosteronism, hypertension, microRNAs, genetic markers, machine learning, serum, primary aldosteronism, diagnostics

## Abstract

**Background:**

Primary aldosteronism (PA) is the principal cause of secondary hypertension; it leads to significantly elevated cardiovascular morbidity and mortality, but only a fraction of its cases ever get detected, partially due to diagnostic procedures that are difficult to perform and to interpret. More straightforward diagnostic methods are needed. Lateralized, or unilateral PA (uPA), is best treated by surgery. Bilateral PA (bPA) is treated medically.

**Aim:**

The aim of our study was to explore microRNA (miRNA) in peripheral blood as markers of PA, uPA and bPA.

**Methods:**

In groups of subjects with primary hypertension (HT, n = 11), bPA (n = 12), and uPA (n = 16), peripheral serum was used for isolation of total RNA, library preparation, and NGS sequencing to achieve a comparative analysis of miRNA expression. Five-fold cross-validation support vector machine learning (ML) models were employed to search for miRNA that could be used as markers of PA and its forms.

**Results:**

In our cohort of patients, the discovered combinations of miRNAs could, with a high level of accuracy, sensitivity, and specificity, characterize the difference between HT and PA, as well as between a combined group of HT + bPA vs. uPA. The differentiating parameters were moderately good for comparison of bPA vs. uPA.

**Conclusion:**

Within our patient cohort, and using ML, the study identified distinctly different miRNA profiles between HT and PA, as well as between bPA and uPA. Further validation studies may lead to the emergence of a new tool for clinical diagnostics of PA.

## 1 Introduction

Primary aldosteronism (PA) is the principal cause of secondary hypertension, where the latest measurements of clinically obvious forms estimate a prevalence of at least 10% in hypertensive patients and above 20% among individuals with resistant hypertension (1–3). The share of the hypertensive population that undergoes screening for PA is below 2%, and, overall, less than 1% of patients with obvious PA get diagnosed (1, 4). Identification of PA in hypertensive patients is important, since untreated non-physiologic aldosterone overproduction per se may provoke substantial target organ damage in addition to the effects of hypertension (1). The considerably higher cardiovascular morbidity and mortality of PA compared to primary (essential) hypertension (HT) accentuates the need for less invasive, more affordable, and more clinically straightforward diagnostic methods (5, 6).

The current diagnostic procedure of PA is complicated and costly, requiring confirmatory testing and invasive adrenal venous sampling for subtyping (7), which partly explains the low detection rates. Unilateral dominant overproduction of aldosterone is best treated with adrenalectomy (8–10). Compared to treatment with mineralocorticoid antagonists (MRA), used in bilateral disease, surgery has been associated with higher cure rates and lower morbidity (10, 11).

MicroRNA (miRNA) is a class of small, double-stranded noncoding RNA that participates in posttranscriptional regulation of up to 60% of human genes (12). MiRNAs have been found to be highly involved in the production and peripheral effects of aldosterone (12, 13). MiRNAs secreted in the circulation remain stable in peripheral blood, which potentially makes them good biomarker candidates. In PA, peripheral blood miRNAs may be altered both due to adrenal cellular changes leading to pathological overproduction of aldosterone, and also as a result of its effects on target tissues (12). Investigations on tissue miRNA have revealed differences between aldosterone-producing adenomas (APAs) and the normal adjacent adrenal gland (14). This raises the potential that PA patients may have a distinct miRNA profile in their circulation.

Unilateral (uPA) and bilateral PA (bPA) seem to have different underlying biology, which might explain potentially divergent microRNA spectrum of these conditions. This divergence could exist even though the majority of cases of uPA may not be represented by APA but by aldosterone-producing nodules (APN) or micronodules (APM) that are also seen in bPA (15, 16). Bilateral PA generally displays milder hormonal derangements than the classical unilateral APAs (1). The major part of lesions in PA have mutations in aldosterone-driver genes, but the types of mutations are different in bPA and uPA (17). The most frequent mutation in APA is in KCNJ5 (and the next most common is in CACNA1D) (17); the most frequent mutation in APM (and in APN) in bPA is in CACNA1D – where the mutation in KCNJ5 is very rare (18). In previous studies, surgical treatment of uPA demonstrated better outcomes than medical treatment of uPA, regardless of whether the underlying histopathological subtype for uPA was a classical APA or other forms such as APM (8, 19). It is important to try to find a marker permitting to differentiate uPA from bPA – thus identifying patients benefitting from expensive and complicated lateralizing procedures, and patients that could bypass those and start medical treatment.

The hormonal derangements in patients with PA can be associated with differences at the miRNA level when compared to individuals with essential hypertension (12). Several miRNAs contribute to the regulation of blood pressure in the renin-angiotensin-aldosterone system (RAAS) (12, 13). In a project studying only cases of known PA, Decmann et al. have shown that bPA and uPA may (at least, to some extent) be differentiated by their miRNA profile (20). In a recent study of multiple markers for endocrine hypertension, miRNAs were shown to help to differentiate between PA and HT (21). This study used 173 miRNAs preselected by the researchers as a part of the analyzed multiomics.

The aim of the present study was to investigate differences in miRNA expression in the peripheral serum of patients with HT, PA, and its subtypes bPA and uPA.

## 2 Materials and methods

Hypertensive patients were evaluated for PA in Karlstad Central Hospital and in Uppsala University Hospital, Sweden, in accordance with the current guidelines (7). Consecutively included patients were divided into groups with primary hypertension - HT (without any form of PA, thus without suppressed renin levels and with normal aldosterone-renin ratio) - and PA, which were further subclassified as uPA or bPA. All patients with confirmed PA had undergone unstimulated adrenal vein sampling. The group of uPA comprised individuals with lateralization index >4, and the group of bPA those with a lateralization index <3. Serum samples from 52 individuals were included in the preliminary quality control analysis, and from them, 39 had a sufficiently high content of total RNA to permit later analysis of miRNA, which led to the subgroups of uPA (n = 16), bPA (n = 12), and HT (n = 11). For the clinical baseline characteristics of the study groups, see **Supplementary Table S1**.

Serum was separated from whole blood under 10 minutes' centrifugation at 1000 g, and frozen to -70 C for storage. The samples were routinely collected when the patients were diagnosed with either HT or PA within our project, under normokalemia. In cases of PA, sampling was performed before initiation of specific medical treatment (by mineralocorticoid receptor antagonists, MRA) or before adrenalectomy.

### 2.1 Nucleic acid extraction and miRNA library sequencing

Total RNA, including miRNA, was isolated using the miRNeasy Serum/Plasma Advanced Kit (Qiagen, Hilden, Germany). RNA quality was evaluated by a TapeStation RNA ScreenTape Analysis (Agilent, Santa Clara, California, USA), and miRNA concentration by a Qubit MicroRNA assay (ThermoFisher Scientific, Waltham, Massachusetts, USA). The QIAseq miRNA library kit (Qiagen) was used to prepare a library from 10 ng of total RNA. NextSeq500/550 (Illumina Inc., San Diego, California, USA) was utilized to do 75 cycles of single-read sequencing to produce at least 10 million reads per sample.

### 2.2 Bioinformatics analysis

MicroRNA-sequencing reads were quality controlled with trimming and adapter removal using cutadapt (22). UMI-tools (23) were used for extracting Unique Molecular Identifiers (UMI-tags) and follow-up read deduplication. Bowtie (24) was used for aligning against the GRCh38 reference genome with the following settings: -n 1 -l 30 –norc –best –strata -m 1. FeatureCounts (25) was used for counting microRNAs aligned to the reference genome matching sequences present in miRbase (26), and DESeq2 (27) was used for differential miRNA expression. A minimum of 20 counts in at least 10 samples were used as expression threshold - based on detection limits generated using qPCR. MiRNA counts in the samples derived from Decmann et al. were generated in the same manner as described above (20).

### 2.3 Quantitative PCR validation

Quantitative PCR validation was used to assess the minimum detection limit and overlapping miRNA expression trends. Out of the 39 of the miRNA samples analyzed by sequencing, 37 were also analyzed using a real-time PCR panel analysis with a miRCURY LNA miRNA PCR Custom panel (QIAGEN Genomic Services). The subgroups encompassed uPA (n = 16), bPA (n = 11), and HT (n = 10). Four spike-in controls were added: a cDNA synthesis control (UniSp6), a DNA spike-in (UniSp3), and two RNA isolation controls (UniSp2, UniSp4) for hemolysis assessment. Samples had a hemolysis ratio of ≤ 7.0, and in case of lack of expression of miRNAs, in those with a maximum of three missing samples mean imputation dependent on sample group adherence was utilized. NormFinder (28) identified hsa-let-7d-5p as a stable candidate for normalization using the sequencing expression data, and was used for reference normalization and calculation of delta Cq (ΔCq).

### 2.4 Statistics

A Wald test with a p-value adjustment (q-value) corrected for multiple testing, according to Benjamini and Hochberg, was used for statistical comparison between two groups in the differential miRNA-seq expression. A likelihood ratio test (LRT) with a p-value adjustment (q-value) corrected for multiple testing, according to Benjamini and Hochberg, was used for comparison between the three groups in the differential miRNA-seq expression. A q-value of less than 0.05 indicates significant differential expression. We adjusted for potential sample origin confounding effects in the statistical model. The Kolmogorov-Smirnov test was used to check for normality in the qPCR analysis, and Welch's t-test (p-value) was used to compare the two groups statistically. Significant differential expression threshold was set at p ≤ 0.05.

## 3 Results

### 3.1 Differential expression of circulating miRNAs

**Figure 1:**
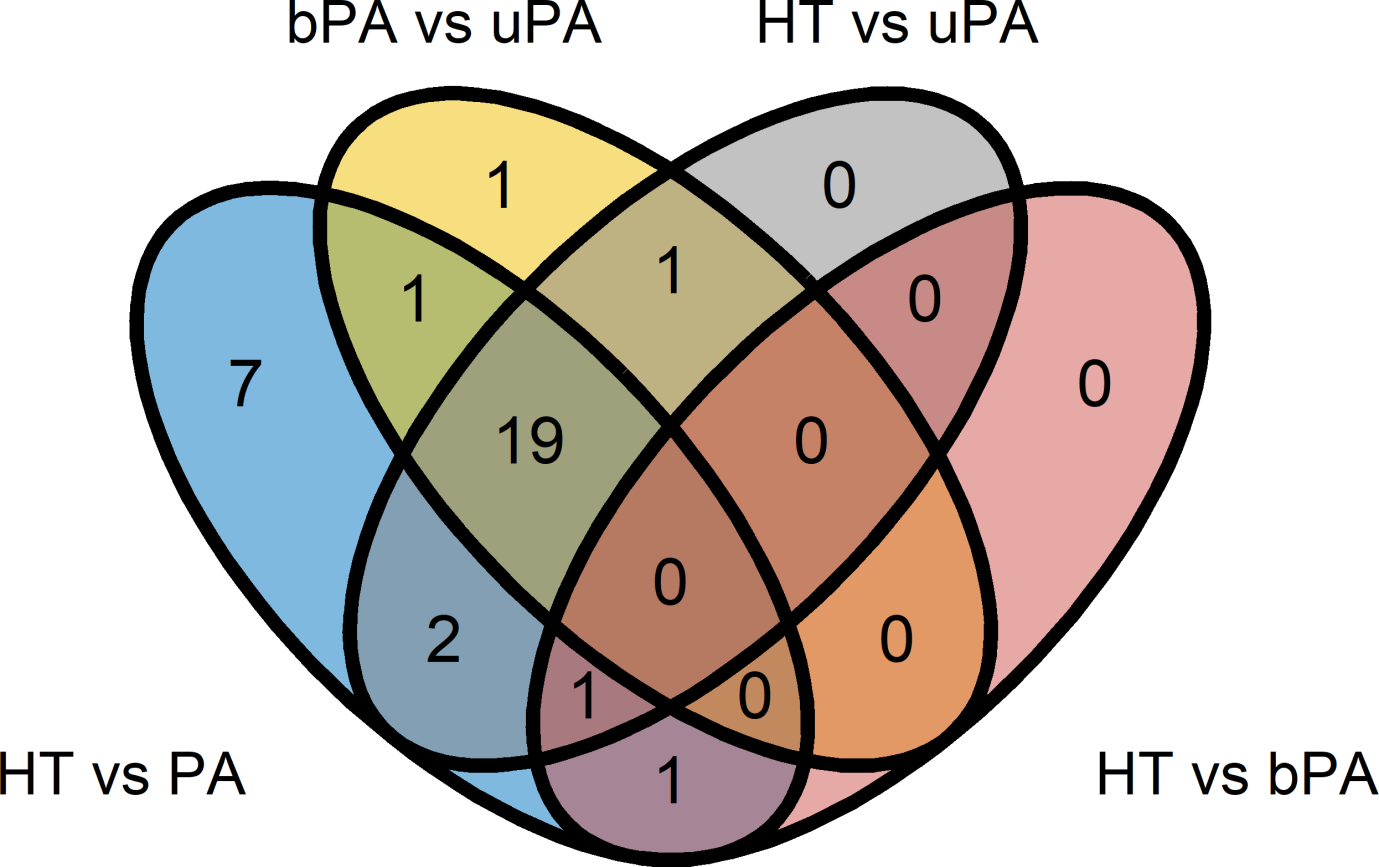
MiRNAs significantly differentially expressed between the study groups. HT, primary hypertension; PA, primary aldosteronism; bPA, bilateral primary aldosteronism; uPA, unilateral primary aldosteronism.

In total, 609 miRNAs could be detected in the samples, but only 81 of them met the criteria for baseline expression based on the limit of detection threshold from qPCR experiments. Out of these, 33 showed a significant expressional difference between the study groups (**Supplementary Table S2**). Further, 31 out of the 33 miRNAs were significantly different between HT and PA (pooled bPA and uPA), but only 7 miRNAs (hsa-miR-15b-5p, hsa-miR-342-3p, hsa-miR-17-5p, hsa-miR-652-3p, hsa-miR-409-3p, hsa-miR-223-5p, and hsa-miR-660-5p) were specific for just that difference (**Figure 1**, **Table 1**). Twenty-three miRNAs differed between HT and uPA, but none of them was recognized as unique (**Figure 1**, **Supplementary Table S2**). Interestingly, there were only two expressional miRNA differences between HT and bPA, with hsa-miR-223-3p sharing a significant overlap with HT vs PA and hsa-miR-625-5p overlapping with all the other three comparisons of HT vs PA, HT vs uPA, and HT vs bPA (**Figure 1**, **Supplementary Table S2**). When comparing uPA and bPA samples, we were able to detect 22 miRNAs with a significant expressional difference, but only one of them was proven to be unique to this comparison (hsa-miR-193b-5p). A summary of unique significant differences is presented in **Table 1**.

**Table 1:** The list of unique differentially expressed (UDE) miRNAs specific for the respective comparisons between the study groups according to **Figure 1**.

Compared study groups	number of UDE miRNAs	Respective UDE miRNAs
HT vs PA	7	hsa-miR-15b-5p, hsa-miR-342-3p, hsa-miR-17-5p, hsa-miR-652-3p. hsa-miR-409-3p, hsa-miR-223-5p, hsa-miR-660-5p
HT vs PA: HT vs bPA	1	hsa-miR-223-3p
bPA vs uPA	1	hsa-miR-193b-5p
HT vs PA: HT vs uPA: HT vs bPA	1	hsa-miR-625-5p
HT vs PA: HT vs uPA	2	hsa-miR-125b-5p, hsa-let-7f-5p
HT vs PA: bPA vs uPA	1	hsa-miR-1224-5p
bPA vs uPA: HT vs uPA	1	hsa-let-7e-5p
HT vs PA: bPA vs uPA: HT vs uPA	19	hsa-miR-10b-5p, hsa-miR-99a-5p, hsa-miR-126-3p, hsa-miR-26b-5p, hsa-miR-92a-3p, hsa-miR-320b, hsa-miR-744-5p, hsa-miR-30e-5p, hsa-miR-378a-3p, hsa-miR-181b-5p, hsa-miR-484, hsa-miR-181a-5p, hsa-miR-21-5p, hsa-miR-193a-5p, hsa-miR-140-5p, hsa-let-7a-5p, hsa-miR-126-5p, hsa-let-7d-5p, hsa-miR-130a-3p
Total	33	

The sign ":" between the subgroups comparisons corresponds to "and".

**Figure 2:**
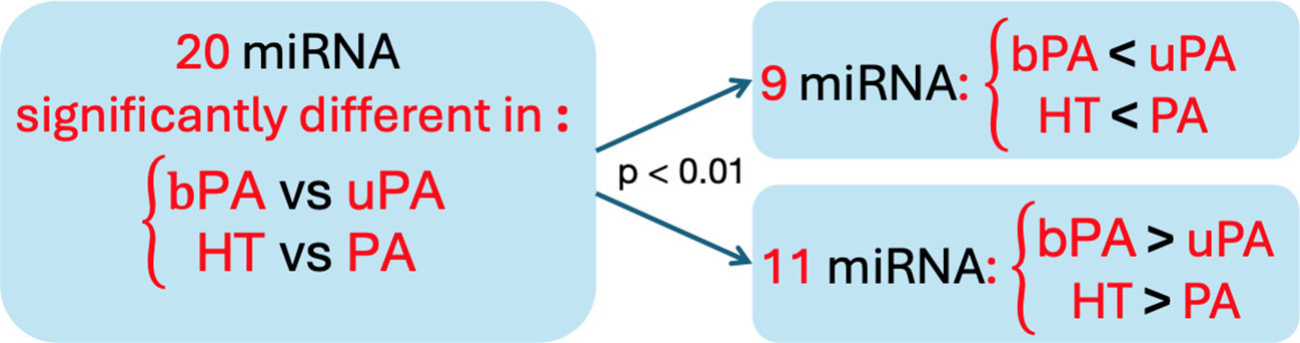
The trend of the similarities of miRNA distribution within the 20 miRNA both significantly different in HT vs. PA and in bPA vs. uPA. HT, primary hypertension; PA, primary aldosteronism; bPA, bilateral primary aldosteronism; uPA, unilateral primary aldosteronism.

We noted an overlap of 20 miRNAs that were significantly different in HT vs. PA and in bPA vs. uPA. Nine of these 20 miRNAs had a significantly lower expression in HT vs. PA as well as in bPA vs. uPA, and 11 of these 20 were vice versa significantly more expressed in HT vs. PA as well as in bPA vs. uPA (Fisher's exact test p < 0.01; **Figure 2**). A similarity between miRNA expression in bPA and HT subgroups could thus be observed.

A description of possible pathophysiological qualities of some of the circulating miRNAs that were significantly different between the groups in our study is summarized in the **Supplementary Table S3**, Supplement. Detailed lists of all miRNAs significantly different between the study groups are presented in **Supplementary T** **able S2**, and the measured differences in their expression levels between the respective groups are presented in **Supplementary Table S4**.

### 3.2 Identification of primary aldosteronism

To explore a possible diagnostic test for PA based on circulating miRNAs, we used a support vector machine (SVM) learning model based on a radial basis function with a 5-fold cross-validation resampling using Caret (28). To discriminate HT from PA samples in our dataset, the machine learning (ML) model used five miRNAs: hsa-miR-10b-5p, hsa-miR-99a-5p, hsa-miR-92a-3p, hsa-miR-30e-5p, and hsa-miR-223-5p – with an accuracy of 100%, an AUC of 1.0, a precision of 1.0, a recall of 1.0, a sensitivity of 1.0, and a specificity of 1.0.

To identify possibly surgically amenable disease, patients with uPA were compared to a combined pool of individuals with HT and bPA. A total of 24 miRNAs were significantly different between these groups. Using five miRNAs in the same ML model: hsa-miR-92a-3p, hsa-miR-10b-5p, hsa-miR-485-5p, hsa-let-7e-5p, and hsa-miR-99a-5p, we could discriminate uPA with a 94% accuracy, an AUC of 0.946, a precision of 0.949, a sensitivity of 0.949, and a specificity of 0.938.

While again using the same ML model, we also explored the possibility of discriminating bPA from uPA, utilizing three miRNAs: hsa-miR-92a-3p, hsa-miR-10b-5p, and hsa-miR-193a-5p. Bilateral PA could be distinguished from uPA with an accuracy of 85%, an AUC of 0.880, a precision of 0.893, a sensitivity of 0.857, and a specificity of 0.875. To validate this, we applied this model to the data from the study by Decmann et al. (20). Cross-validation was performed separately in our samples and in the data from Decmann et al. to control for the confounder related to different centers and even for the fact that we used serum, and Decmann et al. utilized plasma for analyses. With the help of our prediction model, we were able to correctly identify 63% of samples; however, with an AUC of only 0.312, precision of 0.636, sensitivity of 0.633, and specificity of 0.625.

We further evaluated these proposed models, by using both nucleic acid sequencing and polymerase chain reaction. Nineteen of the significant miRNAs discovered in the sequencing results were selected for subsequent qPCR validation. A total of 6 miRNAs maintained the same expression pattern and overlapped significant expression differences: hsa-miR-10b-5p, hsa-miR-21-5p, hsa-miR-30e-5p, hsa-miR-126-5p, hsa-miR-223-3p, and hsa-miR-223-5p (**Table 2**), but the qPCR results alone do not fully cover any of the proposed models (**Supplementary Table S5**). Thus, our ML models could not be verified as applicable to our qPCR results.

**Table 2:** The overlapping, significantly differentially expressed miRNAs between the miRNA-seq and its validation analysis by qPCR (and p-values corresponding to differences of qPCR-results).

Comparisons of the study groups	miRNAs with significant overlap between miRNAseq ^1^ and qPCR ^2^					
	hsa-miR-10b-5p	hsa-miR-21-5p	hsa-miR-30e-5p	hsa-miR-126-5p	hsa-miR-223-3p	hsa-miR-223-5p
HT vs PA	p = 0,0124	p = 0,0133	p = 0,0222	p = 0,02309	p = 0,0019	p = 0,0479
bPA vs uPA				p = 0,0135		
HT vs uPA	p = 0,0094	p = 0,0087	p = 0,029	p = 0,0068		
HT vs bPA					p = 0,0132	
HT+bPA vs uPA	p = 0,0246	p = 0,01607		p = 0,0039		

miRNAseq ^1^ – miRNA sequencing by NGS.

qPCR ^2^ – quantitative polymerase chain reaction.

HT, primary hypertension; PA, primary aldosteronism; bPA, bilateral PA; uPA, unilateral PA; HT+bPA, combined group of both HT and bPA cases; vs, versus.

## 4 Discussion

In the present study, our aim was to investigate circulating miRNAs in PA compared to essential hypertension, and to expand on prior miRNA data differentiating between uPA and bPA. By analyzing all significantly different miRNAs found between the respective patient groups, and by constructing optimal ML models, we could show that miRNAs have the potential to be used as peripheral blood markers of PA and its subtypes in hypertensive patients. Our qPCR data could *per se* validate our sequencing results, even though these data were not applicable to our ML models – most probably, due to the lower sensitivity of qPCR.

Based on our data, two alternative algorithms in a population with hypertension could be envisaged. The initial step in both algorithms is to find patients with PA within the cohort of hypertensive individuals with the help of the 31 miRNAs that significantly differed between the PA and HT groups. The first alternative would be then to identify those with uPA by using the 22 miRNAs found to be significantly differentially expressed between bPA and uPA. The second alternative would be to identify those with uPA by to utilizing the displayed similarity between the groups of HT and bPA – and searching for miRNAs differentially expressed in uPA compared to pooled HT and bPA cases. The overall aim would thus be to select among the hypertensive individuals those with uPA - in order to refer them for localization procedures (adrenal venous sampling or adrenocortical positron emission tomography) and later, potentially, for adrenalectomy.

### 4.1 Identifying PA in a hypertensive population

In the study of Reel et al. (21), an ML model involving several classes of omics – including 173 miRNA – was used to differentiate between types of endocrine hypertension. Among these miRNAs, 27 were included in the model that could single out patients with PA with a balanced accuracy 90%, sensitivity 0,95 and specificity 0,86. The miRNAs alone stood by themselves for a balanced accuracy of 86%, sensitivity 0,95 and specificity 0,76. Between our identified 31 miRNAs there were only three which overlapped with the 27 miRNAs in the study by Reel et al, namely miRNA-342-3p, miRNA-660-5p and miRNA-26b-5p. Of course, in view of the differences in the study protocols, it would be difficult to directly draw any conclusions from this comparison. Interestingly, we could identify all PA cases by applying an ML model using only five miRNAs. If validated in a separate cohort, this may provide a diagnostic aid in PA.

In addition, we noted that a total of 24 miRNAs had a significantly differential expression pattern between uPA and pooled bPA and HT patients. Since uPA may benefit from surgery after a localization procedure, we applied an ML method using the five miRNAs giving the best accuracy. We could identify 94% of uPA patients with a sensitivity of 0.95 and specificity of 0.94. Thus, the number of necessary localization procedures could perhaps be minimized. However, this would need to be validated in a separate cohort.

### 4.2 Differentiating between unilateral and bilateral PA

The study by Decmann et al. (20) described a total of 50 miRNAs significantly different between uPA and bPA. Only the 9 most significant of these were named, and 4 of these 9 were used in a validation experiment without reaching sensitivity or specificity to permit the use in clinical routine. In our analysis, we detected 22 miRNAs with significant expression differences between the bPA and uPA. This list of 22 miRNAs included one miRNA previously described in Decmann, miRNA-30e-5p. In our ML model to differentiate between uPA and bPA, we used the three miRNAs giving the best diagnostic accuracy, in essence a similar approach as between HT and uPA. This model was then applied to an external dataset on PA (20). Unfortunately, this resulted in an AUC of only 0.312, highlighting the inability to compare our data with Decmann's using the previously established SVM model. A reason for that may be that we used serum, while Decmann et al. used plasma as a source for miRNA extraction. Even in the study by Decmann et al, validation experiments (however not involving ML) failed to give a suitable diagnostic accuracy.

Recently, Vékony et al. published a study investigating the prediction of lateralization in PA using machine learning (ML) based on plasma miRNA from adrenal venous sampling (AVS) and from peripheral blood (29). As in the study of Decmann et al. (20), the authors applied miRNA sequencing in their discovery cohort but based it on AVS samples, and used RT-qPCR for validation in peripheral blood samples and in another cohort. They compared their initial results between AVS and peripheral plasma but could not detect any significant expression differences between these two sample origins. Therefore, they continued their research on peripheral blood data for validation in another cohort of peripheral samples, and on the application of deep learning to differentiate bPA from uPA using RT-qPCR results.

None of the miRNAs chosen in ML models overlapped between Vékony's, Decmanns', or our study. In both Vékony's and Decmann's works (where the final laboratory miRNA discovery, validation, and analysis were produced by the same institution), the miRNAs in bPA had higher expression than in uPA, which the authors in Vékony's work speculated could have to do with the higher adrenal volume in bPA. On the other hand, their conclusion was that the miRNA that made the differentiation between bPA and uPA had extra-adrenal origin. The plasma samples used in both of these studies came from many research centers with unavoidably different routines involving PA patients, even if criteria for lateralization of PA were strict.

We attempted to reanalyze this AVS-sample dataset but found that the sequencing repository only contained a portion of the complete read length, prohibiting us from reanalyzing and comparing this dataset. This, together with the fact that we used peripheral serum in our study and that Vékony et al. did not attach the raw data on the significant differences between the AVS samples nor on differences between the peripheral samples, prevented us from being able to make any in-depth comparison of Vékony et al.'s data.

### 4.3 Similarities between groups of HT and bPA

Interestingly, we did not note any unique significantly different circulating miRNAs between bPA and HT. Instead, a particularly similar miRNA expression pattern in HT and in bPA was seen, as shown in **Figure 2**. There were two miRNA that were significantly different between HT and bPA, but both were not unique for that difference. These observations could be due to an eventually underpowered study, but might also point to similarities between these disease entities.

Substantial volume of data has been shown to support the notion that PA should be seen as a continuum of disease, ranging from subclinical normotensive and only biochemically detectable forms, to milder hypertensive variants (mostly caused by bilateral autonomous aldosterone overproduction due to APM and APN), to further forms of progressively severe hypertension and hypokalemia (where APA appear more often) (1). Aldosterone producing micronodules (APM, previously called aldosterone producing cell clusters - APCC) are present in macroscopically intact adrenals from normotensive patients, and may contain mutations in the aldosterone-driver genes (30). The number of APM, and the proportion of APM harboring mutations in aldosterone-driver genes - increase with aging, but even increase in patients with PA (especially in bPA) compared to adrenals from normotensive individuals (30). The predominant type of mutation in APM is the one in CACNA1D (mutations in KCNJ5 are very rare in APM), while KCNJ5 is the most common mutation in APA (17, 30). Theoretically, these underlying histopathologic and genetic differences may be associated to the differences in the microRNA expression.

### 4.4 Arguments for improving diagnostics

Improving diagnostic procedures for PA is regarded as an important aim (31), as also emphasized in the Endocrine Society guidelines (7). The complexity of the current diagnostic work-up and the growing awareness of the unrecognized true prevalence of the pathologic disbalance in the aldosterone secretion among patients with hypertension require simplification of the approach to diagnose and treat PA (7, 31). As suggested, the mildest forms of PA (with late onset, mild hypertension, and relatively low initial aldosterone-renin ratio), which consequently tend to be bilateral (32, 33), are best treated conservatively/medically (1, 31), and these patients may not need to be subjected to invasive and/or costly lateralization procedures. The clinical boundary between bilateral and unilateral PA is often uncertain, but the latter is generally more often represented by higher aldosterone levels and more robust symptoms (33). The discovery of an easily accessible, sensitive, and specific biomarker for both identification of PA among hypertensive individuals, as well as for subtyping between bPA and uPA is of high priority. Besides the named characteristics, the optimal marker should also be affordable and reproducible. An option to use multiple markers, as done in the study of Reel et al. (21), is scientifically tempting but probably too costly if used on a large scale.

As discussed in the reviews on the topic (12, 13), and illustrated in **Supplementary Table S3** (Supplement), a growing amount of published data points to the fact that miRNAs also may have pathophysiological roles in the derangements seen in PA. Considering the scope of our data, the origin of miRNA-markers in our study, which may be adrenal or non-adrenal, cannot be revealed.

## 5 Limitations

Conducting studies on miRNA is difficult. For example, quality of the used serum, how the samples are collected and stored, and how the miRNA is analyzed could lead to different results. Further, interpatient variations such as medication, co-morbidities and level of hypertension may also affect miRNA levels differently, and lower their diagnostic accuracy in a given patient. Additionally, there is uncertainty regarding the similarity of the serum miRNA profile to that of plasma, as evidenced by various studies yielding inconclusive results (34, 35). Unfortunately, using our SVM model for subclassification of PA, we were unable to obtain similar diagnostic accuracy in an external data source as in our discovery cohort. This could perhaps be connected to the previously listed factors.

## 6 Conclusion

The present study identifies distinctly different serum miRNA profiles between HT and PA, as well as between bPA and uPA, indicating that miRNAs may be used as non-invasive biomarkers. Especially our findings of differences between HT and PA *per* se, as well as the identified potential for direct selection of individuals with uPA is a tempting way forward, which could simplify the choice of localization studies and treatment modalities. Further, the noted similarities between the serum miRNA profile of HT and bPA may correspond to less pronounced physiological and genetical differences between HT vs. bPA, compared to HT vs. uPA. Further validation of the study results may deepen the understanding of the clinical role of miRNA-markers in diagnostics of PA.

## Data Availability

All the relevant data is presented within the manuscript and the **Supplementary Material**. Other patient related research data are not shared due to privacy or ethical restrictions.
