# Supplementary material for: Serum microRNAs as peripheral markers of primary aldosteronism
Source: Front Endocrinol (Lausanne). 2025 Mar 20;16:1511096. doi: 10.3389/fendo.2025.1511096 (PMC11967191; doi:10.3389/fendo.2025.1511096)
Supplement: Supplementary file 1 [file DataSheet1.docx]

Supplementary Material

**Table S1. Baseline clinical characteristics of the study groups.** Data presented as Mean $\pm$ SD, % or Median (interquartile range). BMI: body mass index; SBP: systolic blood pressure; DBP: diastolic blood pressure; PAC: plasma aldosterone concentration; DRC: direct renin concentration; ARR: aldosterone-renin ratio; LI: lateralization index; AVS: adrenal vein sampling; MRA: mineralocorticoid receptor antagonists; ACE: angiotensin receptor inhibitor; ARB: angiotensin receptor blocker; TIA – transitory ischemic attack;

| Baseline characteristics | HT (n = 11) | bPA (n = 12) | uPA (n = 16) |
| --- | --- | --- | --- |
| Sex (% men) | 55 | 33 | 64 |
| Age (years) | $56 \pm6$ | $55 \pm$ 9 | 51 $\pm$9 |
| BMI | $29.7 \pm4.5$ | $29.6 \pm6.8$ | 30.7 $\pm$ 4.4 |
| SBP (mmHg) | $140 \pm12$ | $138 \pm14$ | 160 $\pm$ 19 |
| DBP (mmHg) | $87 \pm8$ | $89 \pm10$ | 97 $\pm$ 10 |
| Hypokalemia (%) | 0 | 58 | 75 |
| Serum potassium (mmol/L) | $4.0 \pm0.3$ | $3.8 \pm$ 0.3 | 3,4 $\pm$ 0,5 |
| Serum creatinine (umol/L) | $70 \pm8$ | $72 \pm13$ | 70 $\pm$ 12 |
| PAC (nmol/L) | 133 (113 – 151) | 601 (533 – 865) | 674 (422 – 810) |
| DRC (mIU/L) * | 36 (29 – 38) | 5.1 (2.8 – 10.3) | 3.0 (1.7 – 5.5) |
| ARR (nmol/mIU) | 3.9 (3.5 – 4.3) | 110 (63 – 288) | 178 (118 – 432) |
| Number of antihypertensive medications (n) ** | 1 (1 – 2) | 3 (1 – 3) | 3 (2 – 4) |
| LI at AVS | - | 1.5 (1.1 – 2.0) | 18.6 (6.3 – 28.4) |
| Samples stored at - 70 ^0^C *** (%) | 100 | 100 | 69 |
| Samples taken while on MRA (%) | 0 | 8 | 19 |
| Initial use of MRA (that were paused during PA diagnostics) (%) | 0 | 33 | 19 |
| ACE-inhibitors or ARB (%) | 18 | 67 | 75 |
| Thiazide diuretics (%) | 9 | 50 | 18 |
| Loop diuretics (%) | 0 | 0 | 0 |
| Dihydropyridine calcium blockers (%) | 18 | 58 | 81 |
| Beta-blockers (%) | 9 | 25 | 50 |
| Alpha-1-blockers (%) | 0 | 17 | 31 |
| Verapamil (%) | 0 | 0 | 0 |
| Amilorid (%) | 0 | 8 | 6 |
| Renin inhibitors (%) | 0 | 0 | 0 |
| Imidazoline receptor antagonists (%) | 0 | 0 | 6 |
| Ischemic heart disease **** (%) | 0 | 8 | 6 |
| Chronic heart failure (%) | 0 | 0 | 0 |
| Ischemic / hemorrhagic stroke of TIA (%) | 0 | 0 | 0 |
| Diabetes mellitus or glucose intolerance (%) | 9 | 17 | 13 |
| Sleep apnea syndrom (%) | 27 | 17 | 19 |
| Chronic renal failure (%) | 0 | 0 | 0 |
| Atrial fibrillation (%) | 0 | 8 | 6 |
| Hyperlipidemia and/or statin use (%) | 9 | 33 | 19 |

* - renin values below the lowest assessment limit of 1.6 mIU/L were registered as 1.6 mIU/L

**** -** no one of the study subjects was treated by Torasemid (that may influence miRNA-21 (1))

***** -** the other samples from Uppsala University Hospital were stored at – 20 ^0^C.

**** - history of acute coronary syndrome / angina / percutaneous coronary intervention

**Table S2**: The 33 discovered miRNA significantly different between respective patients groups.

**^1^** - miRNA shown as significantly different even in the study of Decmann et al. (21)

**^2^** - miRNA shown as significantly different even in the study of Reel et al. (22)

| **hsa-miRNA** | **HT vs PA** | **bPA vs uPA** | **HT vs uPA** | **HT vs bPA** | **HT + bPA vs uPA** |
| --- | --- | --- | --- | --- | --- |
| hsa-let-7a-5p | * | * | * |  | * |
| hsa-let-7d-5p | * | * | * |  | * |
| hsa-let-7e-5p |  | * | * |  | * |
| hsa-let-7f-5p | * |  | * |  | * |
| hsa-miR-10b-5p | * | * | * |  | * |
| hsa-miR-1224-5p | * | * |  |  | * |
| hsa-miR-125b-5p | * |  | * |  | * |
| hsa-miR-126-3p | * | * | * |  | * |
| hsa-miR-126-5p | * | * | * |  | * |
| hsa-miR-130a-3p | * | * | * |  | * |
| hsa-miR-140-5p | * | * | * |  | * |
| hsa-miR-15b-5p | * |  |  |  |  |
| hsa-miR-17-5p | * |  |  |  |  |
| hsa-miR-181a-5p | * | * | * |  | * |
| hsa-miR-181b-5p | * | * | * |  | * |
| hsa-miR-193a-5p | * | * | * |  | * |
| hsa-miR-193b-5p |  | * |  |  | * |
| hsa-miR-21-5p | * | * | * |  | * |
| hsa-miR-223-3p | * |  |  | * |  |
| hsa-miR-223-5p | * |  |  |  |  |
| hsa-miR-26b-5p **^2^** | * | * | * |  | * |
| hsa-miR-30e-5p **^1^** | * | * | * |  | * |
| hsa-miR-320b | * | * | * |  | * |
| hsa-miR-342-3p **^2^** | * |  |  |  |  |
| hsa-miR-378a-3p | * | * | * |  | * |
| hsa-miR-409-3p | * |  |  |  |  |
| hsa-miR-484 | * | * | * |  | * |
| hsa-miR-625-5p | * |  | * | * |  |
| hsa-miR-652-3p | * |  |  |  |  |
| hsa-miR-660-5p **^2^** | * |  |  |  |  |
| hsa-miR-744-5p | * | * | * |  | * |
| hsa-miR-92a-3p | * | * | * |  | * |
| hsa-miR-99a-5p | * | * | * |  | * |
| Total: 33 | 31 | 22 | 23 | 2 | 24 |

**Table S3: Some previously described properties of miRNAs that were significantly different between the groups in the present study.** * To simplify the description, the signs > or < here will signify respectively higher or lower expression of a given miRNA in the study groups. HT: primary hypertension, PA: primary aldosteronism, bPA: bilateral primary aldosteronism, and uPA: unilateral primary aldosteronism. ** The miRNA among those chosen in ML-models in the present study.

| Expression differences found in the present study | Name / suffix of the corresponding hsa-miRNA- | Some of the previously described functions of the discussed hsa-miRNA- |
| --- | --- | --- |
| HT > PA * | -125b | -125b is upregulated in cardiac fibroblasts by angiotensin-II, may have a role in cardiac fibrosis development (4). |
| HT > PA  and  bPA > u PA | -21 | - Weakly expressed in normal heart, but upregulated in models of cardiac hypertrophy and heart failure, and in human heart failure (2).  - In a model of PA, -21 became upregulated in left ventricle; downregulation using antagomirs for -21 exacerbated cardiac dysfunction, hypertrophy, and fibrosis (2).  - Genetic ablation of -21 promotes cardiac injury, remodeling and dysfunction (3).  - Aldosterone synthase (key regulator of fibrotic remodeling in atrial fibrillation) upregulates -21. Torasemide (as aldosterone synthase blocker in vitro) lowers -21 (1). |
|  | -181a | -181a targets the renin-gene; suppression of miR-181a in renal cortex of hypertensive patients results in elevated intrarenal renin-production, thus affecting hypertensive mechanisms (5).  - treatment with mimic of -181a had as much hypotensive effect as RAAS-blockade with enalaprilat (6).  - circulating 181a corresponds to renal 181a - colocalized with renin in collecting duct; has pleiotropic blood pressure regulating effects, is positively correlated with blood pressure; can serve as a new biochemical correlate of blood pressure (7).  -181a is a potential protective regulator of Aldosterone–MR-pathway mediated cardiac remodeling (8). |
|  | -181b | - 181b is downregulated in cardiac fibroblasts by angiotensin-II, may have a role in cardiac fibrosis development (4). |
|  | -10b ** | - downregulated in human aldosterone-producing adenoma relative to nontumorous adrenal cortex (9).  - 10b is a hypoxia-inducible microRNA, specifically targeting mRNA of CYP11B1 and CYP11B2 steroidogenic genes in H295R human adrenocortical cells - which thus may be one of mechanisms behind decreasing aldosterone production in hypoxia (10). |
| HT < PA | -223 ** | - circulating -223 was shown as one of the markers of hypertension-induced heart failure, correlating with its progression and with circulating BNP (brain natriuretic peptide) (11).  - elevated levels of circulating -223 predicted cardiovascular death in a cohort of  patients with symptomatic coronary artery disease (12).  - 223 is an abundant circulating endocrine-acting blood cell-produced miRNA that has strong anti-atherogenic effects in the blood vessel wall (13). |
| HT < PA  and  bPA < u PA | -30e ** | - Overexpression of -30e protects podocytes from aldosterone-induced apoptosis and mitochondrial dysfunction – targeting mitochondria-related proapoptotic gene BNIP3L. Inhibition of -30e induces this apoptosis (14). |

**References to Table S3:**

1. Adam O, Zimmer C, Hanke N, Hartmann RW, Klemmer B, Böhm M, et al. Inhibition of aldosterone synthase (CYP11B2) by torasemide prevents atrial fibrosis and atrial fibrillation in mice. J Mol Cell Cardiol. 2015 Aug;85:140–50.

2. Ball JP, Syed M, Marañon RO, Hall ME, Kc R, Reckelhoff JF, et al. Role and Regulation of MicroRNAs in Aldosterone-Mediated Cardiac Injury and Dysfunction in Male Rats. Endocrinology. 2017 Jun 1;158(6):1859–74.

3. Syed M, Ball JP, Mathis KW, Hall ME, Ryan MJ, Rothenberg ME, et al. MicroRNA-21 ablation exacerbates aldosterone-mediated cardiac injury, remodeling, and dysfunction. Am J Physiol-Endocrinol Metab. 2018 Dec 1;315(6):E1154–67.

4. Jiang X, Ning Q, Wang J. Angiotensin II induced differentially expressed microRNAs in adult rat cardiac fibroblasts. J Physiol Sci. 2013 Jan;63(1):31–8.

5. Marques FZ, Campain AE, Tomaszewski M, Zukowska-Szczechowska E, Yang YHJ, Charchar FJ, et al. Gene Expression Profiling Reveals Renin mRNA Overexpression in Human Hypertensive Kidneys and a Role for MicroRNAs. Hypertension. 2011 Dec;58(6):1093–8.

6. Jackson KL, Gueguen C, Lim K, Eikelis N, Stevenson ER, Charchar FJ, et al. Neural suppression of miRNA-181a in the kidney elevates renin expression and exacerbates hypertension in Schlager mice. Hypertens Res. 2020 Nov;43(11):1152–64.

7. Marques FZ, Romaine SPR, Denniff M, Eales J, Dormer J, Garrelds IM, et al. Signatures of miR-181a on the Renal Transcriptome and Blood Pressure. Mol Med. 2015 Jan;21(1):739–48.

8. Garg A, Foinquinos A, Jung M, Janssen‐Peters H, Biss S, Bauersachs J, et al. MiRNA ‐181a is a novel regulator of aldosterone–mineralocorticoid receptor‐mediated cardiac remodelling. Eur J Heart Fail. 2020 Aug;22(8):1366–77.

9. MacKenzie SM, Birch LA, Lamprou S, Rezvanisanijouybari P, Fayad M, Zennaro MC, et al. MicroRNAs in aldosterone production and action. In: Vitamins and Hormones [Internet]. Elsevier; 2024 [cited 2024 Apr 25]. p. 137–63. Available from: https://linkinghub.elsevier.com/retrieve/pii/S0083672923000572

10. Nusrin S, Tong SKH, Chaturvedi G, Wu RSS, Giesy JP, Kong RYC. Regulation of CYP11B1 and CYP11B2 steroidogenic genes by hypoxia-inducible miR-10b in H295R cells. Mar Pollut Bull. 2014 Aug;85(2):344–51.

11. Dickinson BA, Semus HM, Montgomery RL, Stack C, Latimer PA, Lewton SM, et al. Plasma microRNAs serve as biomarkers of therapeutic efficacy and disease progression in hypertension‐induced heart failure. Eur J Heart Fail. 2013 Jun;15(6):650–9.

12. Schulte C, Molz S, Appelbaum S, Karakas M, Ojeda F, Lau DM, et al. miRNA-197 and miRNA-223 Predict Cardiovascular Death in a Cohort of Patients with Symptomatic Coronary Artery Disease. Busson P, editor. PLOS ONE. 2015 Dec 31;10(12):e0145930.

13. Shan Z, Qin S, Li W, Wu W, Yang J, Chu M, et al. An Endocrine Genetic Signal Between Blood Cells and Vascular Smooth Muscle Cells. J Am Coll Cardiol. 2015 Jun;65(23):2526–37.

14. Guo Y, Deng X, Chen S, Yang L, Ni J, Wang R, et al. MicroRNA-30e targets BNIP3L to protect against aldosterone-induced podocyte apoptosis and mitochondrial dysfunction. Am J Physiol-Ren Physiol. 2017 Apr 1;312(4):F589–98.

**Table S4. Comparison of expression of miRNA significantly different between the study groups.**

The study groups that are compared: **HT**- essential hypertension; **PA** - pooled cases of primary aldosteronism; **bPA** - bilateral PA; **uPA** - unilateral PA; **HT+bPA** - a pooled group of HT and bPA. Data presented as: **baseMean** - Average normalized count values; **log2FoldChange** - Effect size estimate as reported on a logarithmic scale to base 2; **IfcSE** - Standard error estimate for the log2 fold change estimate; **p-value**: P-value of the test for the miRNA; **q-value**: adjusted P-value for multiple testing for the miRNA.

| **HT vs PA** | **miRNA** | **baseMean** | **log2FoldChange** | **lfcSE** | **p-value** | **q-value** |
| --- | --- | --- | --- | --- | --- | --- |
| 1 | hsa-miR-10b-5p | 628.7367414 | 1.76228046 | 0.252276837 | 2.84E-12 | 2.30E-10 |
| 2 | hsa-miR-99a-5p | 64.68505667 | 2.141785251 | 0.359891568 | 2.66E-09 | 1.08E-07 |
| 3 | hsa-miR-126-3p | 13698.50692 | -1.03019961 | 0.183805372 | 2.08E-08 | 5.63E-07 |
| 4 | hsa-miR-26b-5p | 4132.936308 | -0.706613594 | 0.139455663 | 4.04E-07 | 8.19E-06 |
| 5 | hsa-miR-92a-3p | 316.9005872 | 1.501035904 | 0.304523976 | 8.26E-07 | 1.34E-05 |
| 6 | hsa-miR-1224-5p | 18.57194565 | 1.925278554 | 0.421697269 | 4.98E-06 | 6.73E-05 |
| 7 | hsa-miR-320b | 3845.716437 | 0.777651306 | 0.176482374 | 1.05E-05 | 0.000121621 |
| 8 | hsa-miR-744-5p | 281.9922358 | -0.992722699 | 0.240330582 | 3.62E-05 | 0.000366253 |
| 9 | hsa-miR-30e-5p | 3317.363855 | -0.468899141 | 0.116311725 | 5.54E-05 | 0.000451899 |
| 10 | hsa-miR-625-5p | 57.43150809 | -0.969858627 | 0.240663016 | 5.58E-05 | 0.000451899 |
| 11 | hsa-miR-378a-3p | 54.27768763 | 0.973900058 | 0.24548502 | 7.27E-05 | 0.000535389 |
| 12 | hsa-miR-181b-5p | 56.82915071 | 1.044382909 | 0.269375884 | 0.000105732 | 0.000713691 |
| 13 | hsa-miR-125b-5p | 17.25344797 | 1.349147556 | 0.352356002 | 0.0001287 | 0.0008019 |
| 14 | hsa-miR-484 | 38.39785098 | 1.582260602 | 0.426399947 | 0.000206652 | 0.001195629 |
| 15 | hsa-miR-181a-5p | 171.3265479 | 1.14139154 | 0.317777143 | 0.000328403 | 0.001662542 |
| 16 | hsa-miR-21-5p | 670.5644625 | 0.851240593 | 0.235967398 | 0.000309221 | 0.001662542 |
| 17 | hsa-miR-193a-5p | 290.409792 | 1.159307895 | 0.32825405 | 0.000412835 | 0.001967039 |
| 18 | hsa-miR-140-5p | 17.54436651 | -1.219225748 | 0.366729164 | 0.000885472 | 0.003984625 |
| 19 | hsa-miR-15b-5p | 884.3024729 | 0.850963046 | 0.260606739 | 0.001093425 | 0.004661442 |
| 20 | hsa-miR-342-3p | 185.1558657 | -0.927154647 | 0.28903229 | 0.001337595 | 0.005417261 |
| 21 | hsa-let-7a-5p | 204.5685624 | -0.834639634 | 0.268036704 | 0.001846317 | 0.007030556 |
| 22 | hsa-miR-126-5p | 3362.289297 | -0.549475563 | 0.177024421 | 0.001909534 | 0.007030556 |
| 23 | hsa-let-7d-5p | 2967.802673 | -0.562246305 | 0.188075665 | 0.002794631 | 0.00943188 |
| 24 | hsa-miR-17-5p | 73.72137036 | -0.810421484 | 0.270027136 | 0.002688654 | 0.00943188 |
| 25 | hsa-miR-130a-3p | 514.1048827 | -0.492514473 | 0.175010635 | 0.004889919 | 0.015843338 |
| 26 | hsa-miR-223-3p | 937.7871115 | -0.979854788 | 0.351694683 | 0.005334724 | 0.016021088 |
| 27 | hsa-miR-652-3p | 358.0166844 | 0.613158791 | 0.220105263 | 0.005340363 | 0.016021088 |
| 28 | hsa-miR-409-3p | 94.85519074 | -0.984110086 | 0.379233025 | 0.009459064 | 0.02736372 |
| 29 | hsa-miR-223-5p | 294.4687778 | -0.492102066 | 0.192257062 | 0.010479131 | 0.029269296 |
| 30 | hsa-miR-660-5p | 156.6367223 | 0.542032455 | 0.222780845 | 0.014973065 | 0.040427276 |
| 31 | hsa-let-7f-5p | 33175.40521 | -0.511349102 | 0.211768521 | 0.015749878 | 0.041152908 |
| **HT+bPA vs uPA** | **miRNA** | **baseMean** | **log2FoldChange** | **lfcSE** | **p-value** | **q-value** |
| 1 | hsa-miR-10b-5p | 628.7367414 | 1.948114949 | 0.314372071 | 5.76E-10 | 4.55E-08 |
| 2 | hsa-miR-99a-5p | 64.68505667 | 2.409016701 | 0.433989878 | 2.84E-08 | 1.12E-06 |
| 3 | hsa-miR-126-3p | 13698.50692 | -1.0916981 | 0.22698278 | 1.51E-06 | 3.98E-05 |
| 4 | hsa-miR-92a-3p | 316.9005872 | 1.682331809 | 0.376470588 | 7.87E-06 | 0.000124344 |
| 5 | hsa-miR-1224-5p | 18.57194565 | 2.275324166 | 0.504256577 | 6.41E-06 | 0.000124344 |
| 6 | hsa-miR-193a-5p | 290.409792 | 1.504497648 | 0.383486337 | 8.74E-05 | 0.00115046 |
| 7 | hsa-miR-320b | 3845.716437 | 0.836182165 | 0.217242828 | 0.000118569 | 0.001338132 |
| 8 | hsa-miR-26b-5p | 4132.936308 | -0.633801749 | 0.171517973 | 0.000219671 | 0.001959978 |
| 9 | hsa-miR-125b-5p | 17.25344797 | 1.555919844 | 0.42153313 | 0.000223289 | 0.001959978 |
| 10 | hsa-miR-181b-5p | 56.82915071 | 1.142054504 | 0.324730921 | 0.000436576 | 0.003448951 |
| 11 | hsa-miR-378a-3p | 54.27768763 | 1.036958698 | 0.309187698 | 0.000797053 | 0.005247265 |
| 12 | hsa-miR-126-5p | 3362.289297 | -0.702778259 | 0.208486377 | 0.000749341 | 0.005247265 |
| 13 | hsa-miR-30e-5p | 3317.363855 | -0.498964941 | 0.153671361 | 0.001166443 | 0.007088384 |
| 14 | hsa-miR-140-5p | 17.54436651 | -1.352278407 | 0.422222038 | 0.001361145 | 0.007680747 |
| 15 | hsa-let-7a-5p | 204.5685624 | -0.970467862 | 0.318136964 | 0.002284821 | 0.012033389 |
| 16 | hsa-miR-181a-5p | 171.3265479 | 1.149418621 | 0.383061064 | 0.002694353 | 0.012520819 |
| 17 | hsa-miR-484 | 38.39785098 | 1.570980547 | 0.522791365 | 0.002655934 | 0.012520819 |
| 18 | hsa-miR-744-5p | 281.9922358 | -0.799240436 | 0.284266855 | 0.004929806 | 0.020643125 |
| 19 | hsa-let-7e-5p | 958.1166177 | -0.902852343 | 0.321378835 | 0.004964802 | 0.020643125 |
| 20 | hsa-let-7d-5p | 2967.802673 | -0.611983095 | 0.22723556 | 0.007077701 | 0.027956919 |
| 21 | hsa-let-7f-5p | 33175.40521 | -0.667158084 | 0.250398325 | 0.007712874 | 0.028688251 |
| 22 | hsa-miR-130a-3p | 514.1048827 | -0.565561202 | 0.213215864 | 0.007989133 | 0.028688251 |
| 23 | hsa-miR-193b-5p | 50.24226748 | 1.2781425 | 0.514479656 | 0.012979174 | 0.044580641 |
| 24 | hsa-miR-21-5p | 670.5644625 | 0.725791017 | 0.297361342 | 0.014655935 | 0.048242451 |
| **HT vs uPA** | **miRNA** | **baseMean** | **log2FoldChange** | **lfcSE** | **p-value** | **q-value** |
| 1 | hsa-miR-10b-5p | 723.5985518 | 1.968448427 | 0.31281555 | 3.12E-10 | 2.28E-08 |
| 2 | hsa-miR-99a-5p | 76.22619173 | 2.432656285 | 0.449764493 | 6.35E-08 | 2.32E-06 |
| 3 | hsa-miR-126-3p | 12633.02426 | -1.073240346 | 0.236979434 | 5.93E-06 | 0.000144322 |
| 4 | hsa-miR-320b | 4091.696706 | 0.860680361 | 0.196024974 | 1.13E-05 | 0.000206231 |
| 5 | hsa-miR-92a-3p | 360.7105995 | 1.700436208 | 0.392116089 | 1.45E-05 | 0.000211303 |
| 6 | hsa-miR-26b-5p | 3836.507829 | -0.611782306 | 0.163593992 | 0.000184285 | 0.002206244 |
| 7 | hsa-miR-125b-5p | 18.53214249 | 1.581383046 | 0.426847079 | 0.000211558 | 0.002206244 |
| 8 | hsa-miR-193a-5p | 305.3208576 | 1.522880286 | 0.421830569 | 0.000305996 | 0.002792211 |
| 9 | hsa-miR-378a-3p | 59.75808584 | 1.057138323 | 0.306459568 | 0.000561585 | 0.004555076 |
| 10 | hsa-miR-181b-5p | 61.64375872 | 1.165096852 | 0.342835329 | 0.000677774 | 0.004947752 |
| 11 | hsa-miR-126-5p | 3305.309836 | -0.673353255 | 0.206204118 | 0.001092828 | 0.007252403 |
| 12 | hsa-miR-30e-5p | 3135.647611 | -0.47711459 | 0.152431656 | 0.001747926 | 0.010551057 |
| 13 | hsa-let-7a-5p | 202.8704893 | -0.946655236 | 0.304515441 | 0.001878955 | 0.010551057 |
| 14 | hsa-miR-181a-5p | 189.9949569 | 1.170142733 | 0.403623046 | 0.00374238 | 0.018212916 |
| 15 | hsa-miR-140-5p | 16.33886368 | -1.335448628 | 0.46025889 | 0.003713616 | 0.018212916 |
| 16 | hsa-miR-484 | 44.81659587 | 1.593184534 | 0.558227539 | 0.004317177 | 0.019697119 |
| 17 | hsa-let-7f-5p | 32939.06371 | -0.650766269 | 0.238772866 | 0.006421165 | 0.026041392 |
| 18 | hsa-let-7d-5p | 2855.380088 | -0.599271348 | 0.218634165 | 0.006125668 | 0.026041392 |
| 19 | hsa-miR-744-5p | 252.4808741 | -0.77272054 | 0.289363669 | 0.007575797 | 0.027196794 |
| 20 | hsa-miR-21-5p | 744.5788552 | 0.758033734 | 0.282140522 | 0.007215653 | 0.027196794 |
| 21 | hsa-let-7e-5p | 1047.927961 | -0.886579675 | 0.333352836 | 0.007823735 | 0.027196794 |
| 22 | hsa-miR-130a-3p | 492.8067307 | -0.537563644 | 0.209451449 | 0.010272143 | 0.034084839 |
| 23 | hsa-miR-625-5p | 47.5437412 | -0.672622436 | 0.266659288 | 0.011655578 | 0.036993792 |
| **bPA vs uPA** | **miRNA** | **baseMean** | **log2FoldChange** | **lfcSE** | **p-value** | **q-value** |
| 1 | hsa-miR-10b-5p | 596.8713401 | 1.957444785 | 0.300858557 | 7.71E-11 | 5.63E-09 |
| 2 | hsa-miR-99a-5p | 64.45458932 | 2.419947924 | 0.45061131 | 7.86E-08 | 2.87E-06 |
| 3 | hsa-miR-126-3p | 12963.4159 | -1.085102851 | 0.243769385 | 8.53E-06 | 0.000207642 |
| 4 | hsa-miR-92a-3p | 288.5723571 | 1.675736253 | 0.393299582 | 2.04E-05 | 0.000320032 |
| 5 | hsa-miR-1224-5p | 19.67007934 | 2.264699644 | 0.533576249 | 2.19E-05 | 0.000320032 |
| 6 | hsa-miR-320b | 3579.485182 | 0.83694692 | 0.221359542 | 0.000156238 | 0.001900895 |
| 7 | hsa-miR-193a-5p | 278.8374512 | 1.521997947 | 0.414560984 | 0.000241274 | 0.002516142 |
| 8 | hsa-miR-26b-5p | 3813.606223 | -0.639895237 | 0.181318439 | 0.000416938 | 0.003804558 |
| 9 | hsa-miR-181b-5p | 53.77368445 | 1.148268034 | 0.335929512 | 0.000630411 | 0.00511333 |
| 10 | hsa-miR-378a-3p | 48.26745239 | 1.044846056 | 0.310313197 | 0.000759716 | 0.005545925 |
| 11 | hsa-miR-30e-5p | 3192.202506 | -0.501681135 | 0.157308449 | 0.00142689 | 0.009469361 |
| 12 | hsa-miR-126-5p | 3137.858003 | -0.697640605 | 0.22346393 | 0.001796646 | 0.010929595 |
| 13 | hsa-miR-140-5p | 16.48409622 | -1.368559829 | 0.447977403 | 0.002250792 | 0.012639062 |
| 14 | hsa-miR-181a-5p | 163.0592396 | 1.156139422 | 0.400603681 | 0.003901808 | 0.020345143 |
| 15 | hsa-miR-484 | 35.28192632 | 1.568586764 | 0.565552487 | 0.005544872 | 0.026985045 |
| 16 | hsa-let-7a-5p | 179.978151 | -0.9676557 | 0.353904571 | 0.006252678 | 0.028527842 |
| 17 | hsa-miR-744-5p | 254.1376538 | -0.798493053 | 0.309518366 | 0.00988598 | 0.040093139 |
| 18 | hsa-miR-21-5p | 606.7885232 | 0.737727943 | 0.284884944 | 0.009609741 | 0.040093139 |
| 19 | hsa-let-7d-5p | 2768.157822 | -0.623016483 | 0.247037097 | 0.011670458 | 0.044839129 |
| 20 | hsa-let-7e-5p | 931.4504662 | -0.903041027 | 0.360879951 | 0.012337857 | 0.045033179 |
| 21 | hsa-miR-193b-5p | 48.16257223 | 1.295723151 | 0.527182503 | 0.013978067 | 0.048590422 |
| 22 | hsa-miR-130a-3p | 489.4848506 | -0.567957333 | 0.232877216 | 0.014733254 | 0.048887615 |
| **HT vs bPA** | **miRNA** | **baseMean** | **log2FoldChange** | **lfcSE** | **p-value** | **q-value** |
| 1 | hsa-miR-223-3p | 1127.375717 | -0.824834343 | 0.247530905 | 0.000861475 | 0.036769623 |
| 2 | hsa-miR-625-5p | 69.48484879 | -0.736913304 | 0.224356044 | 0.001021378 | 0.036769623 |

**Table S5. qPCR miRNA difference between the study groups.**

The study groups that are compared: **HT**- essential hypertension; **PA** - pooled cases of primary aldosteronism; **bPA** - bilateral PA; **uPA** - unilateral PA; **HT+bPA** - a pooled group of HT and bPA. Data presented as p-value from Welch T-test.

| **miRNA** | **HT vs PA** | **bPA vs uPA** | **HT vs uPA** | **HT vs bPA** | **HT+bPA vs uPA** |
| --- | --- | --- | --- | --- | --- |
| hsa-let-7e-5p | 0.02105223 | 0.085168413 | 0.004527964 | 0.208521643 | 0.007039874 |
| hsa-miR-10b-5p | 0.012369785 | 0.101776253 | 0.00935645 | 0.15920748 | 0.024586363 |
| hsa-miR-126-3p | 0.037424232 | 0.097074051 | 0.014394469 | 0.207291171 | 0.016929389 |
| hsa-miR-126-5p | 0.0230889 | 0.013477003 | 0.006780431 | 0.187313737 | 0.003860609 |
| hsa-miR-130a-3p | 0.118100411 | 0.381408315 | 0.159784812 | 0.191117574 | 0.246661091 |
| hsa-miR-130b-3p | 0.094937214 | 0.200796059 | 0.265120631 | 0.055562305 | 0.424824513 |
| hsa-miR-15b-5p | 0.039009489 | 0.093784324 | 0.021091479 | 0.117169333 | 0.015734395 |
| hsa-miR-193a-5p | 0.16209633 | 0.060167292 | 0.062738496 | 0.457957628 | 0.038255562 |
| hsa-miR-20a-5p | 0.045424677 | 0.131870045 | 0.14629958 | 0.030747457 | 0.439562703 |
| hsa-miR-21-5p | 0.013264716 | 0.060461056 | 0.008715461 | 0.123525243 | 0.016060959 |
| hsa-miR-223-3p | 0.001920865 | 0.174295618 | 0.001868601 | 0.013187009 | 0.012780871 |
| hsa-miR-223-5p | 0.047846495 | 0.246261507 | 0.03311598 | 0.173309139 | 0.069357668 |
| hsa-miR-23a-3p | 0.017739059 | 0.284682963 | 0.012065247 | 0.085088616 | 0.043877886 |
| hsa-miR-26b-5p | 0.020971416 | 0.409939494 | 0.028764544 | 0.047121188 | 0.164063898 |
| hsa-miR-30e-5p | 0.022195344 | 0.3604194 | 0.028992744 | 0.0628599 | 0.225514749 |
| hsa-miR-320b | 0.002639801 | 0.099216863 | 0.001392698 | 0.049080116 | 0.00711037 |
| hsa-miR-451a | 0.071494127 | 0.252066096 | 0.047092643 | 0.212536341 | 0.079787606 |
| hsa-miR-92a-3p | 0.013100115 | 0.192484097 | 0.006847353 | 0.104112453 | 0.025038645 |
| hsa-miR-99a-5p | 0.007889588 | 0.090232219 | 0.005225615 | 0.138967282 | 0.01648991 |
